# Unexpected Diversity of *pepA* Genes Encoding Leucine Aminopeptidases in Sediments from a Freshwater Lake

**DOI:** 10.1264/jsme2.ME15117

**Published:** 2016-03-03

**Authors:** Shun Tsuboi, Shigeki Yamamura, Akio Imai, Kazuhiro Iwasaki

**Affiliations:** 1 National Institute for Environmental Studies (NIES), Center for Regional Environmental Research305–8506, TsukubaJapan; 2 National Institute for Environmental Studies (NIES), Center for Environmental Biology and Ecosystem Studies305–8506, TsukubaJapan

**Keywords:** leucine aminopeptidase gene, sediments, hypereutrophic freshwater lake, genetic diversity, prokaryotic community

## Abstract

We herein designed novel PCR primers for universal detection of the *pepA* gene, which encodes the representative leucine aminopeptidase gene, and investigated the genetic characteristics and diversity of *pepA* genes in sediments of hypereutrophic Lake Kasumigaura, Japan. Most of the amino acid sequences deduced from the obtained clones (369 out of 370) were related to PepA-like protein sequences in the M17 family of proteins. The developed primers broadly detected *pepA*-like clones associated with diverse bacterial phyla—*Alpha*-, *Beta*-, *Gamma*-, and *Deltaproteobacteria*, *Acidobacteria*, *Actinobacteria*, *Aquificae*, *Chlamydiae*, *Chloroflexi*, *Cyanobacteria*, *Firmicutes*, *Nitrospirae*, *Planctomycetes*, and *Spirochetes* as well as the archaeal phylum *Thaumarchaeota*, indicating that prokaryotes in aquatic environments possessing leucine aminopeptidase are more diverse than previously reported. Moreover, prokaryotes related to the obtained *pepA*-like clones appeared to be *r*- and *K*-strategists, which was in contrast to our previous findings showing that the neutral metalloprotease gene clones obtained were related to the *r*-strategist genus *Bacillus*. Our results suggest that an unprecedented diversity of prokaryotes with a combination of different proteases participate in sedimentary proteolysis.

The bacterial enzymatic hydrolysis of proteins (proteolysis) has a prominent influence on nitrogen cycling in lake sediments. The hydrolysis of particulate proteins, which are representative components of the particulate organic matter in sediments (15), is often the rate-limiting step in this protein degradation (4, 5), and the subsequent deamination of amino acids by microbes may strongly contribute to nitrogen regeneration in sediments (11). However, our knowledge of the distribution and diversity of bacterial proteases in sediments is limited.

Extracellular proteases are generally secreted from intact living cells and play a significant role in the proteolysis of particulate proteins outside cells. In natural environments, intracellular (cytosolic) proteases, which are released by cell death or lysis, may also be responsible for this proteolysis. For example, Nannipieri *et al.* (29) reported that some enzymes liberated by cell death and lysis, particularly those associated with humic substances and minerals, maintained their activity for an unexpectedly long time. Alkaline metalloprotease, neutral metalloprotease (Npr), and serine protease, which are secreted from living cells (10, 31), are representative extracellular proteases derived from bacteria (13). Phylogenetic analyses of the genes encoding these proteases have been conducted in some environments (26–28, 33), and *npr*-related genes were identified in the sediments of a hypereutrophic lake (38). Since the occurrence of these genes is related to the dominance of the genus *Bacillus* and high interstitial ammonium concentrations in sediments, proteolysis by sedimentary bacteria has been suggested to play an important role in nitrogen regeneration (38).

Leucine aminopeptidases belong to the M1 and M17 protease families (22). One of the genes encoding the M17 family of leucine aminopeptidases, *pepA*, has been detected in bacterial isolates including *Escherichia coli* (35) and *Rickettsia prowasekii* (40). Although leucine aminopeptidases are generally regarded as intracellular enzymes (17), a recent study identified a *pepA* gene that encodes a secretory leucine aminopeptidase (16). Thus, the subcellular location of these leucine peptidases remains to be clarified.

Leucine aminopeptidases appear to be significant proteolytic agents in aquatic environments. This enzymatic activity has been detected in lake water (5, 9, 14), groundwater (37), river water (12, 37), intertidal mudflat sediments (23), inlet sediments (30), and lake sediments (6). Furthermore, a previous study reported that all 44 bacterial strains isolated from marine environments exhibited positive leucine aminopeptidase activity, but with marked differences in activity levels among strains (20). However, information on the diversity of bacteria possessing leucine aminopeptidases or the occurrence of functional genes encoding these enzymes in natural aquatic environments is limited.

The aims of the present study were 1) to develop a *pepA*-specific universal PCR primer set for the detection of leucine aminopeptidase-harboring bacteria; 2) to evaluate the applicability of the designed primers; and 3) to investigate the genetic characteristics and diversity of *pepA* genes in the sediments of a hypereutrophic lake.

## Materials and Methods

### Bacterial strains and culture conditions

As representative organisms for evaluating the applicability of the newly designed primer pair, we used pure cultures of *E. coli* JM109 and *Pseudomonas stutzeri* IFO3773 because other strains of both species are known to possess *pepA* genes. *E. coli* JM109 and *P. stutzeri* IFO3773 were cultured in Luria-Bertani medium and medium containing (L^−1^) 10 g polypeptone, 2 g yeast extract, and 1 g MgSO_4_·7H_2_O (pH 7.0), respectively, at 37ºC.

### Design of primers

**Fig. 1:**
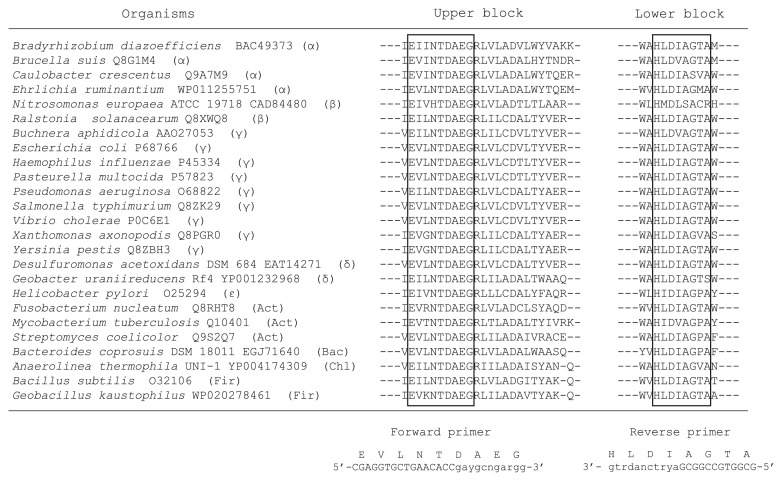
Alignment of PepA partial amino acid sequences and consensus amino acid sequences used to design primers for *pepA*. “α”, “β”, “γ”, “δ”, “ɛ”, “Act”, “Bac”, “Chl” and “Fir” indicate *Alpha*-, *Beta*-, *Gamma*-, *Delta*-, *Epsilon*-*proteobacteria*, *Actinobacteria*, *Bacteroides*, *Chloroflexi*, and *Firmicutes*, respectively.

PCR primers were designed from the alignment of the amino acid sequences encoded by the leucine aminopeptidase gene in 25 bacterial species (Fig. 1). In order to design the primers, we applied the consensus-degenerate hybrid oligonucleotide primers (CODEHOP) strategy (http://blocks.fhcrc.org/codehop.html) (32). The parameters for designing the primers were an annealing temperature ≤60ºC and primer degeneracies ≤128.

### Collection of sediment core samples and DNA extraction from pure cultures and sediment samples

The environmental samples used in the present study were freshwater lake sediments from Lake Kasumigaura, Japan. An acrylic tube (30 cm in length, internal diameter=4 cm) was used as a gravity corer in order to collect sediments at the center of Lake Kasumigaura (36º01′57″N, 140º24′25″E). Eight cores were collected at each sampling event. The sediment cores were transported to the laboratory. In the present study, we used sections at a depth of 4–6 cm in the sediment cores collected bimonthly between February and December 2007. Bacterial DNA was extracted from pure cultures and sediment samples using a FastDNA Pro Soil Kit (Q-Biogene, Carlsbad, CA, USA) according to the manufacturer’s protocol.

### PCR conditions

In order to detect the *pepA* gene, PCR was performed in a volume of 10 μL containing 1×PCR buffer (with MgCl_2_), 0.2 mM of each dNTP, 1 μM of each primer, 0.05 U TaKaRa *Ex Taq* (TaKaRa Bio, Otsu, Japan), and the DNA sample. The touchdown PCR program was as follows: initial denaturation at 95ºC for 5 min followed by 35 cycles of denaturation at 94ºC for 1 min, annealing for 1 min, and extension at 72ºC for 1 min. The annealing temperature decreased from 65ºC to 60ºC at 0.5ºC cycle^−1^ for the first 10 cycles and was kept constant at 60ºC for the last 25 cycles. The PCR reaction was performed using the thermal cycler TaKaRa Thermal Cycler Dice Gradient or TaKaRa Thermal Cycler Dice Touch (TaKaRa Bio). In order to determine whether this primer pair had the ability to amplify the *pepA* genes, the genomic DNAs of *E. coli* JM109 and *P. stutzeri* IFO3773 were used as positive controls.

### Clone library construction, sequencing, and phylogenetic analysis

Clone libraries were constructed using the February and August samples from a depth of 4–6 cm. The amplified *pepA* genes were cloned into the pMD20-T vector with the Mighty-TA Cloning Kit (TaKaRa Bio) according to the manufacturer’s instructions. The constructed vectors were transformed into *E. coli* JM109 competent cells (TaKaRa Bio). The transformed *E. coli* JM109 was cultured on a Luria-Bertani plate containing ampicillin (100 μg mL^−1^), 5-bromo-4-chloro-3-indolyl-β-D-galactopyranoside (80 μg mL^−1^), and isopropyl- β-D-thiogalactopyranoside (100 μM) at 37ºC overnight and distinguished by blue–white selection. The white colonies were checked for the presence of an insert fragment of the correct size by direct PCR using the vector primers M13 primer M4 and M13 primer RV. More than 180 *E. coli* JM109 colonies with a PCR fragment of the correct size were randomly selected for each environmental sample and used in further sequencing analyses. Positive fragments were sequenced using BigDye Terminator Kit v. 3.1 (Applied Biosystems, Carlsbad, CA, USA) and the vector primers described above, and sequences were determined on an Applied Biosystems 3730 DNA Analyzer. Distance matrices were calculated based on the DNADIST program in PHYLIP (PHYLogeny Inference Package) 3.695 (http://evolution.genetics.washington.edu/phylip.html) and were used to group the obtained sequences into operational taxonomic units (OTUs) with a distance cut-off of 0.3 using the software Mothur (34). Rarefaction curves were calculated using R version 2.15.2 statistical software (R Development Core Team; http://www.r-project.org/). Evolutionary distance dendrograms were constructed by the neighbor-joining method with the MEGA 6 software package (36). Confidence in the dendrogram topology was evaluated using a bootstrap analysis with 1,000 resamplings.

### Real-time quantitative PCR (qPCR) assay

The copy numbers of *pepA* genes were quantified in sediment samples. Standard samples (554 bp) were constructed from the transformed vector including the *P. stutzeri* IFO3773-derived *pepA* gene in *E. coli* JM109, which were amplified using M13 primer M4 and M13 primer RV. The standard samples generated by PCR were purified using the PureLink Quick PCR Purification Kit (Life Technologies, Grand Island, NY, USA) and the single band was visually confirmed by electrophoresis through a 2.0% agarose gel containing 0.5 mg L^−1^ ethidium bromide. The concentrations and copy numbers of the standard DNA samples were measured and calculated using the Quant-it dsDNA Broad-Range Assay Kit and a Qubit Fluorometer (Invitrogen, San Diego, CA, USA) according to the manufacturer’s specifications. qPCR was performed using TaKaRa Thermal Cycler Dice Real-Time System Single and MightyAmp for Real Time (SYBR Plus) (TaKaRa Bio) according to the manufacturer’s protocol. All analyses were conducted in triplicate with an appropriate dilution of each extracted DNA sample. The qPCR thermal program was as follows: initial denaturation at 98ºC for 2 min, followed by 45 cycles of 98ºC for 10 s, 64ºC for 15 s, and 68ºC for 1 min. The specificity of the qPCR products was checked by 1.5% agarose gel electrophoresis. In order to quantify *pepA* genes in the sediment samples, environmental DNA samples were diluted to reduce the influence of inhibitors on PCR amplification.

### Nucleotide sequence accession numbers

The partial *pepA* gene sequences retrieved have been deposited in the DDBJ/EMBL/GenBank databases under the following accession numbers: LC048882 for *E. coli* JM109, LC048883 for *P. stutzeri* IFO3773, and LC048512–LC048881 for environmental clones.

## Results

### Primer design and amplification of *pepA* genes from pure cultures

**Fig. 2:**
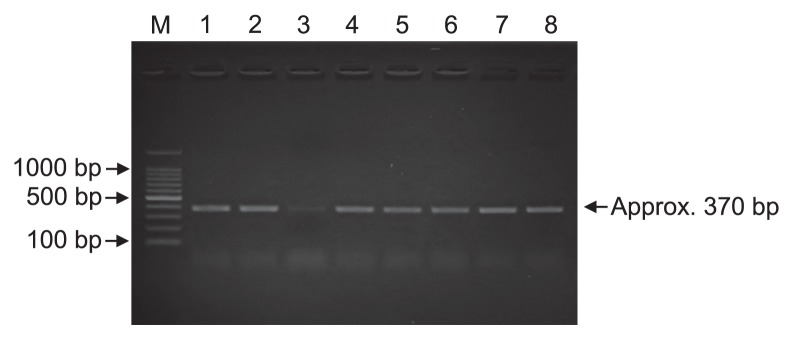
Detection of *pepA* genes from pure cultures and sediment samples. Lanes 1–6: PCR products obtained from DNA in sediment samples from February, April, June, August, October, and December, respectively. Lane 7: PCR products from the genomic DNA of *Escherichia coli* JM109. Lane 8: PCR products from the genomic DNA of *Pseudomonas stutzeri* IFO3773.

In PepA sequences of approximately 500 amino acids, the CODEHOP program identified only two conserved regions as possible PCR primers: “EVLNTDAEG” and “HLDIAGTA” (Fig. 1). Based on the sequences in these regions, two CODEHOPs were designed: pepAf-codehop (5′-CGAGGT GCTGAACACCGAYGCNGARGG-3′) and pepAr-codehop (5′-GCGGTGCCGGCGAYRTCNADRTG-3′). The designed primer set successfully amplified the genomic DNA of *E. coli* JM109 and *P. stutzeri* IFO3773 at the expected length of approximately 370 bp without any extra bands (Fig. 2). Sequencing showed that the translated amino acid sequence from the amplicon of *E. coli* JM109 was closely related to the amino acid sequence of a protein from the cytosol aminopeptidase family from *E. coli* 4-203-08_S3_C3 (accession no. KEK90327) with a similarity value of 100%. The translated amino acid sequence from the *pepA* amplicon derived from *P. stutzeri* IFO3773 also showed a high similarity (100%) to multifunctional aminopeptidase A from *P. stutzeri* ATCC 14405 (accession no. WP003284686).

### Diversity and sequence analysis of *pepA* genes from freshwater lake sediments

The designed primer pair successfully amplified genes with the predicted size (approximately 370 bp) from the sediments of Lake Kasumigaura without any extra bands (Fig. 2). Of these PCR amplicons, the February and August samples were cloned, sequenced, and then phylogenetically analyzed; 182 clones from February and 188 clones from August were analyzed. The 75 OTUs from February and 69 OTUs from August were grouped into a clone library with 118 OTUs at a similarity cut-off value of 70%. Multiple diversity indices on the *pepA* gene clone libraries are presented in Table 1. Shannon-Weaver (*H*′) and the reciprocal of Simpson diversity indices (1/*D*) of the retrieved *pepA* clones were both slightly higher in February than in August. This difference was also supported by rarefaction curves of the *pepA* clone libraries (Fig. S1).

We performed an alignment of the residues surrounding the catalytic amino acid residue (Fig. S2). All of the retrieved sequences possessed the catalytic amino acid residue of leucine aminopeptidases: arginine (“R”). Moreover, the amino acid residues in the retrieved PepA sequences next to the catalytic amino acid residue, which may influence leucine aminopeptidase activity (3), were of diverse types: histidine (“H”), isoleucine (“I”), leucine (“L”), methionine (“M”), glutamine (“Q”), and valine (“V”).

**Fig. 3:**
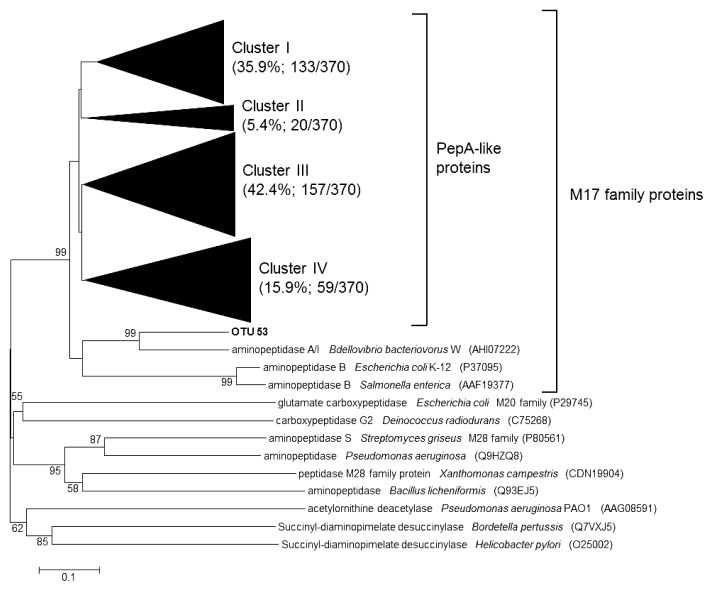
Evolutionary distance dendrogram of retrieved PepA sequences with reference sequences of M20 and M28 family proteins from the NCBI database based on OTU groupings. Bootstrap values less than 50% are not shown.

An evolutionary distance dendrogram was generated incorporating reference sequences from the NCBI database (http://www.ncbi.nlm.nih.gov/) based on partial amino acid sequences after removing the primer sequences from the retrieved clones (approximately 109 amino acids) (Fig. 3). All of the sequences retrieved by PCR showed similarities to the amino acid sequences of aminopeptidases belonging to the M17 family of proteins in the NCBI database and were apparently different from other zinc-dependent proteases in the M28 and M20 families of proteins. Moreover, all sequences were phylogenetically close to PepA-like proteins, except for one clone. The PepA clones retrieved were close to those of various bacterial phyla, including the *Alpha*-, *Beta*-, *Gamma*-, and *Deltaproteobacteria*, *Acidobacteria*, *Actinobacteria*, *Aquificae*, *Chlamydiae*, *Chloroflexi*, *Cyanobacteria*, *Firmicutes*, *Nitrospirae*, *Planctomycetes*, and *Spirochetes* as well as the archaeal phylum *Thaumarchaeota* (Table S1). The major genotypes of *pepA* from February were similar to those from August (Table S1). The genera and families affiliated with the obtained PepA sequences showed a large range of 16S rRNA gene copy numbers per cell (Table S1).

### Phylogenetic analysis of PepA from sediments of a hypereutrophic lake

We used the Basic Local Alignment Search Tool (BLAST) to find matching *pepA* sequences in the GenBank database. Table 2 presents phylogenetic information for the representative 10 *pepA* OTUs. Detailed phylogenetic trees were also generated (Fig. S3). Most of the PepA sequences retrieved in this study were assigned to four clusters: cluster I (35.9% of sequences; 133 out of 370), cluster II (5.4%; 20 out of 370), cluster III (42.4%; 157 out of 370), and cluster IV (15.9%; 59 out of 370) (Fig. 3). Gammaproteobacterial methanotroph-related *pepA* clones were dominant in cluster I (11.6%; 43 out of 370), particularly OTU 6, related to the cytosol aminopeptidase of *Methylocaldum szegediense*, and OTU 7, multifunctional aminopeptidase A of *Methylococcus capsulatus* (Fig. S3a). These sequences had 81–88% amino acid similarities to the cytosol aminopeptidase of *M. szegediense* (Table 2). The second most abundant clones in this cluster were those assigned to OTU 2, which were related to the aminopeptidase of “*Candidatus* Competibacter denitrificans” and the hypothetical protein of “*Ca.* Contendobacter odensis” (7.0%; 26 out of 370) (Fig. S3a). Cluster II contained the fewest sequences of the four clusters (Fig. S3b). In cluster III, the most abundant sequences (16.2%; 60 out of 370) were associated with the clones related to the hypothetical protein of “*Ca.* Magnetobacterium sp. MYR-1” (65–73% similarities) (Fig. S3c and Table 2). The clones evolutionally close to *Chloroflexi*-related aminopeptidase (65–82% similarities) were also abundant (9.7%; 36 out of 370) (Fig. S3c). The most abundant clones in cluster IV (5.4%; 20 out of 370) were those related to class *Dehalococcoidia* (66–83% similarities) (Fig. S3d).

**Fig. 4:**
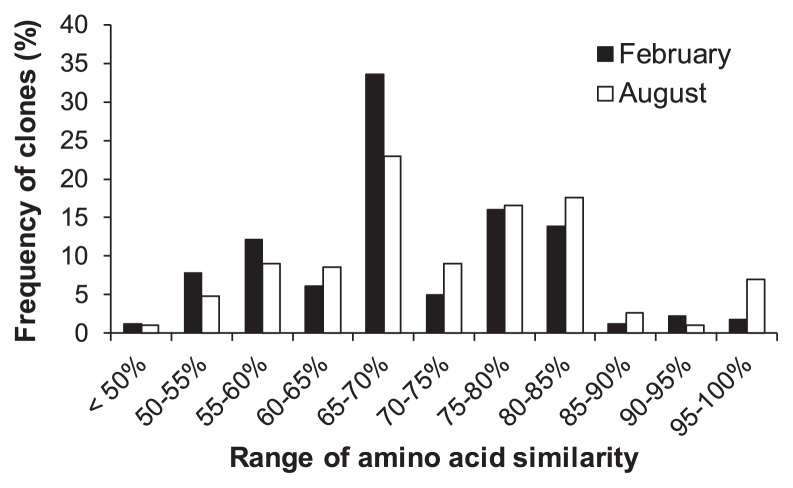
Frequency distribution of similarities between amino acid sequences deduced from retrieved *pepA* genes and those in the GenBank database.

At the amino acid level, the sequences retrieved from the sediments had similarities to NCBI database entries of 49–96% in February and 49–100% in August (Fig. 4). The similarity range of 65–70% had the most amino acid sequences as deduced from *pepA* clones in both February and August. There was also a large number of PepA sequences with low similarities (<65%) to the database sequences. These results indicate that most of the sedimentary PepA sequences were unknown and different from the PepA sequences derived from pure cultures currently in the databases.

### Quantification of *pepA* genes in sediments of a hypereutrophic lake

qPCR assays were used to quantify *pepA* gene copy numbers in the sediments (at a depth of 4–6 cm) of Lake Kasumigaura in February and August 2007. The standard curve constructed from the *pepA* gene of *P. stutzeri* IFO3773 was strongly linear (*r*^2^=0.99), ranging from 1.18×10^2^ to 1.18×10^8^ copies per reaction. qPCR amplification efficiency was 85.3%. The copy numbers of *pepA* genes were 1.24×10^8^ (±3.07×10^7^) mL^−1^ sediment in February and 7.20×10^7^ (±2.17×10^7^) mL^−1^ sediment in August, indicating the absence of a significant difference between the sampling periods in *pepA* gene abundance at a depth of 4–6 cm in Lake Kasumigaura sediments.

## Discussion

In the present study, we attempted to develop a *pepA-*specific universal primer pair. We demonstrated the applicability of the designed primers for environmental samples by investigating the diversity and phylogeny of this gene in sediments from a freshwater lake.

Of the two conserved regions of PepA sequences proposed for primers by the CODEHOP program (Fig. 1), the first region (EVLNTDAEG), which was used to design the forward primer, is a very important amino acid sequence for the functioning of PepA. The sixth (aspartic acid; “D”) and eighth amino acid (glutamic acid; “E”) are zinc-binding residues (2). Although the second region (HLDIAG) was shown to be highly conserved in PepA-like protein sequences in previous studies (3, 17), the function of this amino acid motif is not yet known. In the amplicons generated using the designed primers, the catalytic residue arginine (R) was also found in all the clones retrieved (Fig. S2).

The designed primers permitted the specific detection of the expected amplicons without any extra bands, both from pure cultures and sedimentary DNA (Fig. 2). The results of a phylogenetic analysis indicated that most of the retrieved sequences were related to PepA-like protein sequences in the M17 protein family (Fig. 3). Therefore, the designed primers may be specifically useful for detecting *pepA* genes. The designed primers have the ability to broadly detect putative *pepA* genes from diverse phyla including *Acidobacteria*, *Aquificae*, *Chlamydiae*, *Cyanobacteria*, *Nitrospirae*, *Planctomycetes*, and *Spirochetes*, which were not used to design CODEHOPs (Table S1). A *pepA* clone related to an uncultured marine *Thaumarchaeota* was also obtained (Fig. S3d). These results suggest that *pepA* genes are widely distributed in diverse taxonomic groups of prokaryotes in sediments. Unidentified or hardly cultivable microorganisms (*e.g.*, *Candidatus* species) may also be significant contributors to leucine aminopeptidase production in sediments because most of the amino acid sequences deduced from the *pepA* clones retrieved had low identity to those of PepA sequences from pure cultures in the GenBank database (Fig. 4). Thus, the primers that we developed may be a powerful culture-independent tool for examining the community structure of leucine aminopeptidase–producing bacteria and investigating the expression of the *pepA* gene.

A phylogenetic analysis of the *pepA* genes found in the present study showed an abundance of those derived from various bacterial groups having specific functions such as methane oxidation (*M. szegediense*) (25) and glycogen accumulation (“*Ca.* Competibacter denitrificans”) (24) (Fig. S3a). Moreover, we also found a high abundance of PepA close to the class *Dehalococcoidia*, which are anaerobic bacteria (18) (Fig. S3d). These results indicate that aerobic and anaerobic microbes may both be relevant to PepA production and that diverse functional groups possess proteolytic functions in sediments. Leucine aminopeptidase activity has been associated with heterotrophic bacteria in aquatic environments on the basis of the strong correlation detected between bacterial secondary production and leucine aminopeptidase activity (5, 39). In the present study, we found *pepA* clones similar to *Sideroxydans lithotrophicus* and *Gallionella* sp., which are chemolithoautotrophic bacteria (1, 19) (Fig. S3a). Thus, our results suggest a markedly higher diversity of prokaryotes possessing leucine aminopeptidase in aquatic environments than previously suspected. The copy numbers of *pepA* genes obtained in the present study appear to be similar to those of functional genes responsible for inorganic nitrogen metabolism such as *nirS*, *nirK*, and *amoA*, which range from approximately 10^6^ to 10^8^ copies g^−1^ of dry sediment (21); however, the quantification of functional genes with CODEHOPs may be somewhat inaccurate because of differences in the priming efficiencies of primers to the respective target sequences (7). This implies that *pepA*-mediated organic nitrogen metabolism plays a significant role in nitrogen cycles.

We previously reported that the temporally synchronous occurrence of 16S rRNA and *npr*-related genes from the genus *Bacillus* in the same sediment samples used in the present study positively correlated with increased interstitial ammonium concentrations (38). This finding suggests that proteolysis by Npr from the genus *Bacillus* is one of the contributors to ammonium production. However, in that study, the relative abundance of the genus *Bacillus* markedly increased in August, whereas ammonium concentrations increased gradually between April and December (38). Thus, the detection of *pepA* genes in this study may provide an important insight into understanding this apparent contradiction; that is, proteases other than Npr (*e.g.*, PepA) may also act to increase ammonium concentrations.

Fierer *et al.* (8) previously reported that *r*-strategists are copiotrophs with high 16S rRNA copy numbers per cell (>5) and large nutritional requirements, whereas *K*-strategists are oligotrophs with low 16S rRNA copy numbers per cell (<2) that prefer low nutrient conditions. Based on this characterization, *r*- and *K*-strategists both appear to possess PepA in Lake Kasumigaura because the candidate contributors of PepA in this study have a wide range of 16S rRNA gene copy numbers per cell (Table S1). In contrast, most of the Npr sequences obtained were phylogenetically related to Npr of the genus *Bacillus*, which is a representative *r*-strategist that increased in August in Lake Kasumigaura (38). The sediment in August had higher nutrient concentrations (dissolved organic carbon, ammonium, dissolved total nitrogen, and orthophosphates) than those in February (38). These results suggest that proteolysis by leucine aminopeptidase is a universal function in aquatic sediments, regardless of season and nutrient conditions. Therefore, PepA may play a particularly important role in decomposing particulate proteins during the early stage of relatively low inorganic nitrogen conditions, and appears to act as a trigger for subsequent proteolysis by Npr. These results indicate that proteolysis by sedimentary bacteria is a ubiquitous process, but one that arises from a complex combination of different types of protease-producing microbial communities depending on environmental conditions.

## Conclusion

We herein developed a novel universal PCR primer set for the detection of leucine aminopeptidase genes (*pepA*) and investigated the genetic characteristics and diversity of *pepA* genes in sediments of a hypereutrophic lake. Our results show that the designed primers have the ability to broadly detect putative *pepA* genes derived from diverse phyla. The diversity of prokaryotes possessing *pepA* in the sediments was markedly higher than previously identified, and *pepA* genes were detected across all seasons and nutrient conditions in the sediments. Our results indicate that proteolysis by sedimentary bacteria is a ubiquitous process, but arises from a complex combination of different types of protease-producing microbial communities.

## Supplementary Material



## Figures and Tables

**Table 1:** Diversity indices for *pepA* clones obtained from sediments of Lake Kasumigaura, Japan.

Month	No. of clones	No. of OTUs	Shannon-Weaver (*H*′)	Simpson (1/*D*)	Coverage
February	182	75	3.86	37.2	0.736
August	188	69	3.74	29.8	0.739

**Table 2:** Phylogenetic information for 10 representative *pepA* OTUs from sediments of Lake Kasumigaura, Japan

OTU	Cluster	No. of clones		Closest BLAST match	Accession No.	Amino acid identity (%)

		Feb	Aug			
OTU 1	III	26	27	hypothetical protein *Candidatus* Magnetobacterium sp. MYR-1	WP040334930	65–73
OTU 2	I	12	13	aminopeptidase A *Candidatus* Competibacter denitrificans Run_A_D11	CDI00939	75–83
			1	hypothetical protein *Candidatus* Contendobacter odensis	WP034430259	85
OTU 3	III	8	5	leucyl aminopeptidase *Chloroflexus aurantiacus* J-10-fl	YP001636914	66–74
		2	7	cytosol aminopeptidase *Chloroflexus* sp. MS-G	WP031458570	67–74
		1	1	cytosol aminopeptidase *Chloroflexus aggregans*	WP012616296	65–67
		1		cytosol aminopeptidase *Roseiflexus* sp. RS-1	WP011955470	68
OTU 4	I	7	13	hypothetical protein OT06_52005 *Candidatus* Thiomargarita nelsonii	KHD04775	74–78
			1	leucyl aminopeptidase *Beggiatoa* sp. PS	EDN67650	78
OTU 5	III	11	7	leucyl aminopeptidase *Candidatus* Nitrospira defluvii	WP041186815	54–58
		1	1	cytosol aminopeptidase *Syntrophus aciditrophicus* SB	WP041584835	59–61
OTU 6	I	5	15	cytosol aminopeptidase *Methylocaldum szegediense*	WP026610414	81–88
OTU 7	I	7	8	cytosol aminopeptidase *Methylocaldum szegediense*	WP026610414	81–84
OTU 8	I		9	leucyl aminopeptidase *Pseudomonas* sp. GM16	WP007909047	98–100
OTU 9	II	3	4	aminopeptidase A *Caldilinea aerophila*	WP014434738	55–57
			1	cytosol aminopeptidase *Roseiflexus* sp. RS-1	WP011955470	54
OTU 10	IV	2	1	cytosol aminopeptidase *Persephonella* sp. IF05-L8	WP029520707	51
		2		leucyl aminopeptidase *Sanguibacter keddieii*	WP012865512	52
		1		cytosol aminopeptidase *Sulfurihydrogenibium* sp. YO3AOP1	WP012458779	50
		1		hypothetical protein Dehalococcoidia bacterium SCGC AB-539-J10	WP029474841	53

## References

[1] Beckwith CR, Edwards MJ, Lawes M, Shi L, Butt JN, Richardson DJ, Clarke TA (2015). Characterization of MtoD from *Sideroxydans lithotrophicus*: a cytochrome c electron shuttle used in lithoautotrophic growth. Front Microbiol.

[2] Burley SK, David PR, Sweet RM, Taylor A, Lipscomb WN (1992). Structure determination and refinement of bovine lens leucine aminopeptidase and its complex with bestatin. J Mol Biol.

[3] Chi MC, Huang HB, Liu JS, Wang WC, Liang WC, Lin LL (2006). Residues threonine 346 and leucine 352 are critical for the proper function of *Bacillus kaustophilus* leucine aminopeptidase. FEMS Microbiol Lett.

[4] Chróst RJ, Chróst RJ (1991). Environmental control of the synthesis and activity of aquatic microbial ectoenzymes. Microbial Enzymes in Aquatic Environments.

[5] Chróst RJ (1992). Significance of bacterial ectoenzymes in aquatic environments. Hydrobiol.

[6] Das B, Nordin R, Mazumder A (2008). Aminopeptidase activity in lake sediments. Verh Internat Verein Limnol.

[7] De Long SK, Kinney KA, Kirisits MJ (2010). qPCR assays to quantify genes and gene expression associated with microbial perchlorate reduction. J Microbiol Met.

[8] Fierer N, Bradford MA, Jackson RB (2007). Toward an ecological classification of soil bacteria. Ecol.

[9] Hałemejko GZ, Chróst RJ (1986). Enzymatic hydrolysis of proteinaceous particulate and dissolved material in an eutrophic lake. Arch Hydrobiol.

[10] Häse CC, Finkelstein RA (1993). Bacterial extracellular zinc-containing metalloproteases. Microbiol Rev.

[11] Herbert RA (1999). Nitrogen cycling in coastal marine ecosystems. FEMS Microbiol Rev.

[12] Hosen JD, McDonough OT, Febria CM, Palmer MA (2014). Dissolved organic matter quality and bioavailability changes across an urbanization gradient in headwater streams. Environ Sci Technol.

[13] Kalisz HM (1988). Microbial proteinases. Adv Biochem Eng Biotechnol.

[14] Kiersztyn B, Siuda W, Chróst RJ (2012). Persistence of bacterial proteolytic enzymes in lake ecosystems. FEMS Microbiol Ecol.

[15] Li H, Minor EC (2015). Biogeochemical characteristics of settling particulate organic matter in Lake Superior: A seasonal comparison. Org Geochem.

[16] Liew SM, Tay ST, Puthucheary SD (2013). Enzymatic and molecular characterization of leucine aminopeptidase of *Burkholderia pseudomallei*. BMC Microbiol.

[17] Lin LL, Hsu WH, Wu CP, Chi MC, Chou WM, Hu HY (2004). A thermostable leucine aminopeptidase from *Bacillus kaustophilus* CCRC 11223. Extremophiles.

[18] Löffler FE, Yan J, Ritalahti KM, Adrian L, Edwards EA, Konstantinidis KT, Müller JA, Fullertton H, Zinder SH, Spormann AM (2013). *Dehalococcoides mccartyi* gen. nov., sp. nov., obligately organohalide-respiring anaerobic bacteria relevant to halogen cycling and bioremediation, belong to a novel bacterial class, *Dehalococcoidia* classis nov., order *Dehalococcoidales* ord. nov. and family *Dehalococcoidaceae* fam. nov., within the phylum *Chloroflexi*. Int J Syst Evol Microbiol.

[19] Lütters-Czckalla S (1990). Lithoautotrophic growth of the iron bacterium *Gallionella ferruginea* with thiosulfate or sulfide as energy source. Arch Microbiol.

[20] Martinez J, Smith DC, Steward GF, Azam F (1996). Variability in ectohydrolytic enzyme activities of pelagic marine bacteria and its significance for substrate processing in the sea. Aquat Microb Ecol.

[21] Martins G, Terada A, Ribeiro DC, Corral AM, Brito AG, Smets BF, Nogueira R (2011). Structure and activity of lacustrine sediment bacteria involved in nutrient and iron cycles. FEMS Microbiol Ecol.

[22] Matsui M, Fowler JH, Walling LL (2006). Leucine aminopeptidases: diversity in structure and function. Biol Chem.

[23] Mayer LM (1989). Extracellular proteolytic enzyme activity in sediments of an intertidal mudflat. Limnol Oceanogr.

[24] McIlroy SJ, Albertsen M, Andresen EK, Saunders AM, Kristiansen R, Stokholm-Bjerregaard M, Nielsen KL, Nielsen PH (2014). ‘*Candidatus* Competibacter’-lineage genomes retrieved from metagenomes reveal functional metabolic diversity. ISME J.

[25] Medvedkova KA, Khmelenina VN, Suzina NE, Trotsenko YA (2009). Antioxidant systems of moderately thermophilic methanotrophs *Methylocaldum szegediense* and *Methylococcus capsulatus*. Microbiol.

[26] Mrkonjic Fuka M, Engel M, Gattinger A, Bausenwein U, Sommer M, Munch JC, Schloter M (2008). Factors influencing variability of proteolytic genes and activities in arable soils. Soil Biol Biochem.

[27] Mrkonjic Fuka M, Engel M, Haesler F, Welzl G, Munch JC, Schloter M (2008). Diversity of proteolytic community encoding for subtilisin in an arable field: spatial and temporal variability. Biol Fertil Soils.

[28] Mrkonjic Fuka M, Engel M, Hagn A, Munch JC, Sommer M, Schloter M (2009). Changes of diversity pattern of proteolytic bacteria over time and space in an agricultural soil. Microb Ecol.

[29] Nannipieri P, Sequi P, Fusi P, Piccolo A (1996). Humus and enzyme activity. Humic Substances in Terrestrial Ecosystems.

[30] Patel AB, Fukami K, Nishijima T (2001). Extracellular proteolytic activity in the surface sediment of a eutrophic inlet. Microb Environ.

[31] Power SD, Adams RM, Wells JA (1986). Secretion and autoproteolytic maturation of subtilisin. Proc Natl Acad Sci USA.

[32] Rose TM, Henikoff JG, Henikoff S (2003). CODEHOP (COnsensus-DEgenerate Hybrid Oligonucleotide Primer) PCR primer design. Nucleic Acids Res.

[33] Sakurai M, Suzuki K, Onodera M, Shinano T, Osaki M (2007). Analysis of bacterial communities in soil by PCR-DGGE targeting protease genes. Soil Biol Biochem.

[34] Schloss PD, Westcott SL, Ryabin T (2009). Introducing mothur: open-source, platform-independent, community-supported software for describing and comparing microbial communities. Appl Environ Microbiol.

[35] Stirling CJ, Colloms SD, Collins JF, Szatmari G, Sherratt DJ (1989). *xerB*, an *Escherichia coli* gene required for plasmid ColE1 site-specific recombination, is identical to *pepA* encoding aminopeptidase A, a protein with substantial similarity to bovine lens leucine aminopeptidase. EMBO J.

[36] Tamura K, Stecher G, Peterson D, Filipski A, Kumar S (2013). MEGA6: Molecular evolutionary genetics analysis version 6.0. Mol Biol Evol.

[37] Tiquia SM (2011). Extracellular hydrolytic enzyme activities of the heterotrophic microbial communities of the Rouge River: an approach to evaluate ecosystem response to urbanization. Microb Ecol.

[38] Tsuboi S, Yamamura S, Imai A, Satou T, Iwasaki K (2014). Linking temporal changes in bacterial community structures with the detection and phylogenetic analysis of neutral metalloprotease genes in the sediments of a hypereutrophic lake. Microb Environ.

[39] Vives Rego J, Billen G, Fontigny A, Somville M (1985). Free and attached proteolytic activity in water environments. Mar Ecol Prog Ser.

[40] Wood DO, Solomon MJ, Speed RR (1993). Characterization of the *Rickettsia prowazekii pepA* gene encoding leucine aminopeptidase. J Bacteriol.

