# Supplementary material for: Unexpected Diversity of pepA Genes Encoding Leucine Aminopeptidases in Sediments from a Freshwater Lake
Source: Microbes Environ. 2016 Mar 3;31(1):49–55. doi: 10.1264/jsme2.ME15117 (PMC4791116; doi:10.1264/jsme2.ME15117)
Supplement: Supplementary file 1 [file 31_49_s1.pdf]

Supplemental materials

Unexpected diversity of *pepA* genes encoding leucine aminopeptidases in sediments from a freshwater lake

Shun Tsuboi[1,2]*, Shigeki Yamamura[1], Akio Imai[1] and Kazuhiro Iwasaki[1]

Affiliation:
[1]National Institute for Environmental Studies (NIES), Center for Regional Environmental Researches, 305-8506, Tsukuba, Japan.
[2]National Institute for Environmental Studies (NIES), Center for Environmental Biology and Ecosystem Studies, 305-8506, Tsukuba, Japan.

***Corresponding author:** Shun Tsuboi
E-mail address: tsuboi.shun@nies.go.jp
Tel: +81-29-850-2204

Table S1. Summary of the best BLAST matches for the obtained PepA-like proteins, and 16S rRNA gene copy numbers per cell, along with the affiliated genus/family

Domain	Phylum (subdivision)	Genus/Family	Similarity (%)	16S rRNA gene copy numbers[a]	Number of clones	
					February	August
Bacteria	*Alphaproteobacteria*	*Azorhizobium*	81	3		1
		Hyphomicrobium	78	1	1	
		Methyloferula	73	n.d.		1
		Phaeospirillum	75	n.d.		1
		Stappia	82	n.d.	1	
	Betaproteobacteria	*Azovibrio*	64	n.d.		1
		Chitinimonas	61	n.d.	1	
		Gallionella	85-86	3		2
		Methylophilus	81	1	1	
		Methylotenera	82-96	2-3	2	
		Methylovorus	63-79	2		2
		Neisseria	59-61	4	1	1
		Nitrosomonas	94	1	1	
		Sideroxydans	90	2	2	
		Sulfuricella	96	2		2
		Thiobacillus	84-97	2	2	1
		Uliginosibacterium	75	n.d.		1
	Gammaproteobacteria	*Arenimonas*	91	n.d		2
		Beggiatoa	78	n.d.		1
		Cellvibrio	78	3	3	1
		Competibacter	76-80	n.d.	12	13
		Hahella	69	5		1
		Marinobacter	80-81	3	1	1
		Methylocaldum	80-88	n.d.	9	25
		Methylococcus	81-83	2	3	2
		Methylosarcina	92	n.d.	1	
		Pseudohaliea	80	n.d.		1
		Pseudomonas	98-100	4-7		9
		Tatlockia	50	n.d.		1
		Thioalkalivibrio	75	1	3	
		Thiomargarita	74-78	n.d.	7	13
	Deltaproteobacteria	*Desulfobulbus*	56	2	1	1
		Desulfococcus	68	1		1
		Desulfosarcina	62	n.d.		1
		Geobacter	61-65	2-4		3
		Smithella	67	n.d.	1	

Table S1. Continuation

Domain	Phylum (subdivision)	Genus/Family	Similarity (%)	16S rRNA gene copy numbers[a]	Number of clones	
					February	August
		Syntrophobacter	68	2		1
		Syntrophus	74	1	3	2
	Acidobacteria	*Acidobacterium*	61	1		1
		Solibacter	73-79	2	4	
	Actinobacteria	*Corynebacterium*	60-61	3-7	2	
		Rubrobacter	61-63	1	1	1
		Sanguibacter	52	4	3	
	Aquificae	*Persephonella*	51	2	2	1
		Sulfurihydrogenibium	50	2-3	1	
	Chlamydiae	*Simkania*	52	1	1	
	Chloroflexi	*Caldilinea*	55-63	2	4	6
		Chloroflexus	70-82	3	13	16
		Dehalococcoidia	72-83	1	16	6
		Herpetosiphon	64	5	1	
		Roseiflexus	54-68	2	1	1
	Cyanobacteria	*Scytonema*	60	n.d.	1	
	Firmicutes	*Bacillus*	100	6-15		1
		Clostridium	49	1-15	1	
		Megasphaera	49	7	1	
		Zymophilus	50	n.d.	1	
	Nitrospirae	*Magnetobacterium*	72-73	n.d.	30	30
		Nitrospira	86-88	1	13	8
	Planctomycetes	*Blastopirellula*	69	n.d.	1	
		Isosphaera	62	3		1
		Pirellula	70-74	1-2	2	2
		Rhodopirellula	60	1	1	
		Scalindua	53-66	n.d.	4	2
		Schlesneria	80	n.d.	1	
		Singulisphaera	56	8	1	
	Spirochetes	*Leptospira*	56-58	2	2	
Archaea	*Thaumarchaeota*	*Nitrosopumilus*	51	1		1

[a]The 16S rRNA gene copy numbers shown are the values in the rRNA database (http://rrndb.umms.med.umich.edu/)

Figure legends

Fig. S1. Rarefaction curves obtained for the *pepA* gene sequences in clone libraries of sediment samples from Lake Kasumigaura. Samples were taken at depths of 4–6 cm in sediment cores collected in February and August 2007. The y-axis shows the number of OTUs grouped at 70% similarity.

Fig. S2. Amino acid sequences surrounding the catalytic sequences of each OTU. The "R"s (arginine; diamond) show the catalytic amino acid residues. The box highlights amino acid residues next to the catalytic amino acid residues that are reported to influence leucine aminopeptidase activity (1). a) OTU 1 to OTU 60, and b) OTU 61 to OTU 118.

Fig. S3. Evolutionary distance dendrograms of amino acid sequences deduced from *pepA* genes in a) cluster I, b) cluster II, c) cluster III, and d) cluster IV. Boxes indicate the representative 10 *pepA* OTUs from the sediments of Lake Kasumigaura. Symbols are used to distinguish different clone libraries: closed circles, February; open squares, August. Numbers to the right of the symbols indicate the number of sequences within the respective clone libraries. Bootstrap values below 50% are not shown.

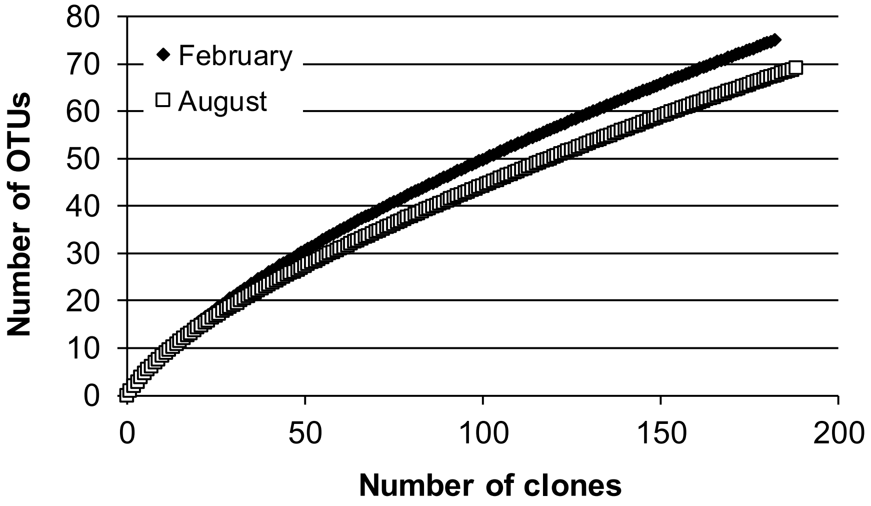

80
70
60
50
40
30
20
10
0
February
August
Number of OTUs
0
50
100
150
200
Number of clones

Fig. S1.

a)

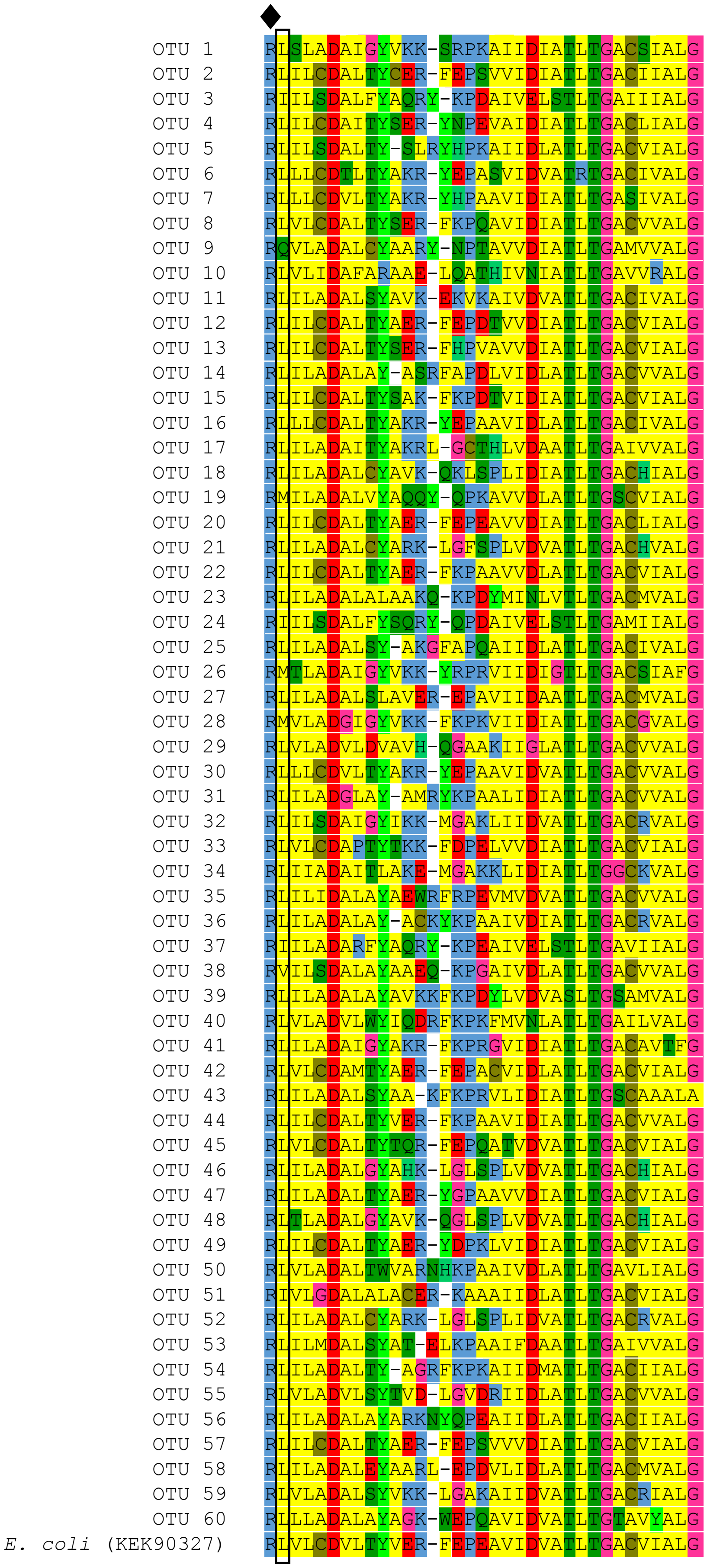
OTU 1 RLSLADAIGYVKK-SRPKAIIDIATLTGACSIALG
OTU 2 RLILCDALTYCER-FEPSVVIDIATLTGACIIALG
OTU 3 RIILSDALFYAQRY-KPDAIVELSTLTGAIIIALG
OTU 4 RLILCDAITYSER-YNPEVAIDIATLTGACLIALG
OTU 5 RLILSDALTY-SLRYHPKAIIDLATLTGACVIALG
OTU 6 RLLLCDTLTYAKR-YEPASVIDVATRTGACIVALG
OTU 7 RLLLCDVLTYAKR-YHPAAVIDIATLTGASIVALG
OTU 8 RLVLCDALTYSER-FKPQAVIDIATLTGACVVALG
OTU 9 RQVLADALCYAARY-NPTAVVDIATLTGAMVVALG
OTU 10 RLVLIDAFARAAE-LQATHIVNIATLTGAVVRALG
OTU 11 RLILADALSYAVK-EKVKAIVDVATLTGACIVALG
OTU 12 RLILCDALTYAER-FEPDTVVDIATLTGACVIALG
OTU 13 RLILCDALTYSER-FHPVAVVDIATLTGACVIALG
OTU 14 RLILADALAY-ASRFAPDLVIDLATLTGACVVALG
OTU 15 RLILCDALTYSAK-FKPDTVIDIATLTGACVIALG
OTU 16 RLLLCDALTYAKR-YEPAAVIDLATLTGACIVALG
OTU 17 RLILADAITYAKRL-GCTHLVDAATLTGAIVVALG
OTU 18 RLILADALCYAVK-QKLSPLIDIATLTGACHIALG
OTU 19 RMILADALVYAQQY-QPKAVVDLATLTGSCVIALG
OTU 20 RLILCDALTYAER-FEPEAVVDIATLTGACLIALG
OTU 21 RLILADALCYARK-LGFSPLVDVATLTGACHVALG
OTU 22 RLILCDALTYAER-FKPAAVVDLATLTGACVIALG
OTU 23 RLILADALALAAKQ-KPDYMINLVTLTGACMVALG
OTU 24 RIILSDALFYSQRY-QPDAIVELSTLTGAMIIALG
OTU 25 RLILADALSY-AKGFAPQAIIDLATLTGACIVALG
OTU 26 RMTLADAIGYVKK-YRPRVIIDIGTLTGACSIAFG
OTU 27 RLILADALSLAVER-EPAVIIDAATLTGACMVALG
OTU 28 RMVLADGIGYVKK-FKPKVIIDIATLTGACGVALG
OTU 29 RLVLADVLDVAVH-QGAAKIIGLATLTGACVVALG
OTU 30 RLLLCDVLTYAKR-YEPAAVIDVATLTGACVVALG
OTU 31 RLILADGLAY-AMRYKPAALIDIATLTGACVVALG
OTU 32 RLILSDAIGYIKK-MGAKLIIDVATLTGACRVALG
OTU 33 RLVLCDAPTYTKK-FDPELVVDIATLTGACVIALG
OTU 34 RLIIADAITLAKE-MGAKKLIDIATLTGGCKVALG
OTU 35 RLILIDALAYAEWRFRPEVMVDVATLTGACVVALG
OTU 36 RLILADALAY-ACKYKPAAIVDIATLTGACRVALG
OTU 37 RIILADARFYAQRY-KPEAIVELSTLTGAVIIALG
OTU 38 RVILSDALAYAAEQ-KPGAIVDLATLTGACVVALG
OTU 39 RLILADALAYAVKKFKPDYLVDVASLTGSAMVALG
OTU 40 RLVLADVLWYIQDRFKPKFMVNLATLTGAILVALG
OTU 41 RLILADAIGYAKR-FKPRGVIDIATLTGACAVTFG
OTU 42 RLVLCDAMTYAER-FEPACVIDLATLTGACVIALG
OTU 43 RLILADALSYAA-KFKPRVLIDIATLTGSCAAALA
OTU 44 RLILCDALTYVER-FKPAAVIDIATLTGACVVALG
OTU 45 RLVLCDALTYTQR-FEPQATVDVATLTGACVIALG
OTU 46 RLILADALGYAHK-LGLSPLVDVATLTGACHIALG
OTU 47 RLILADALTYAER-YGPAAVVDIATLTGACVIALG
OTU 48 RLTLADALGYAVK-QGLSPLVDVATLTGACHIALG
OTU 49 RLILCDALTYAER-YDPKLVIDIATLTGACVIALG
OTU 50 RLVLADALTWVARNHKPAAIVDLATLTGAVLIALG
OTU 51 RIVLGDALALACER-KAAAIIDLATLTGACVIALG
OTU 52 RLILADALCYARK-LGLSPLIDVATLTGACRVALG
OTU 53 RLILMDALSYAT-ELKPAAIFDAATLTGAIVVALG
OTU 54 RLILADALTY-AGRFKPKAIIDMATLTGACIIALG
OTU 55 RLVLADVLSYTVD-LGVDRIIDLATLTGACVVALG
OTU 56 RLILADALAYARKNYQPEAIIDLATLTGACIIALG
OTU 57 RLILCDALTYAER-FEPSVVVDIATLTGACVIALG
OTU 58 RLILADALEYAARL-EPDVLIDLATLTGACMVALG
OTU 59 RLVLADALSYVKK-LGAKAIIDVATLTGACRIALG
OTU 60 RLLLADALAYAGK-WEPQAVIDVATLTGTAVYALG
E. coli (KEK90327) RLVLCDVLTYVER-FEPEAVIDVATLTGACVIALG

Fig. S2.

b)

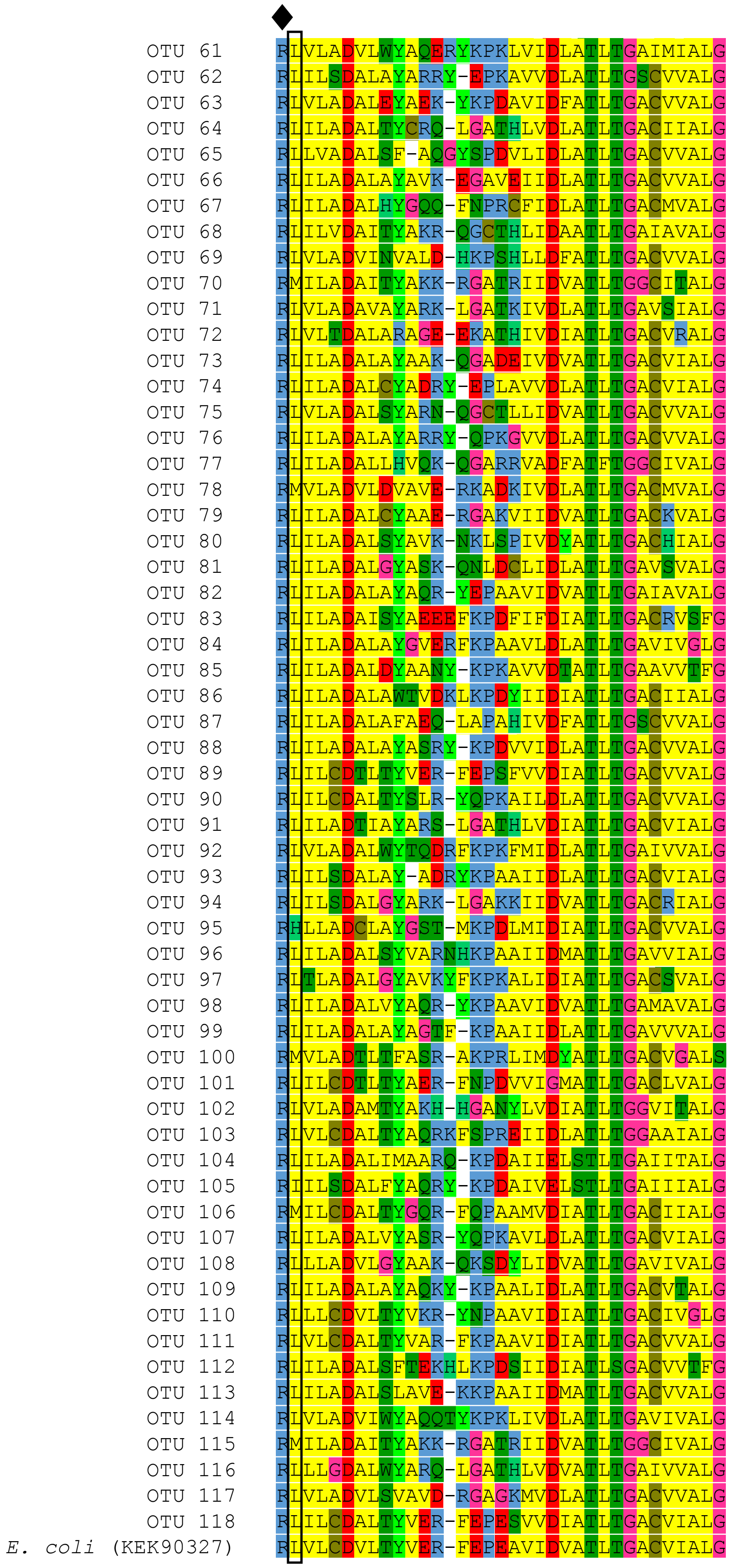

Fig. S2. Continuation.

a)

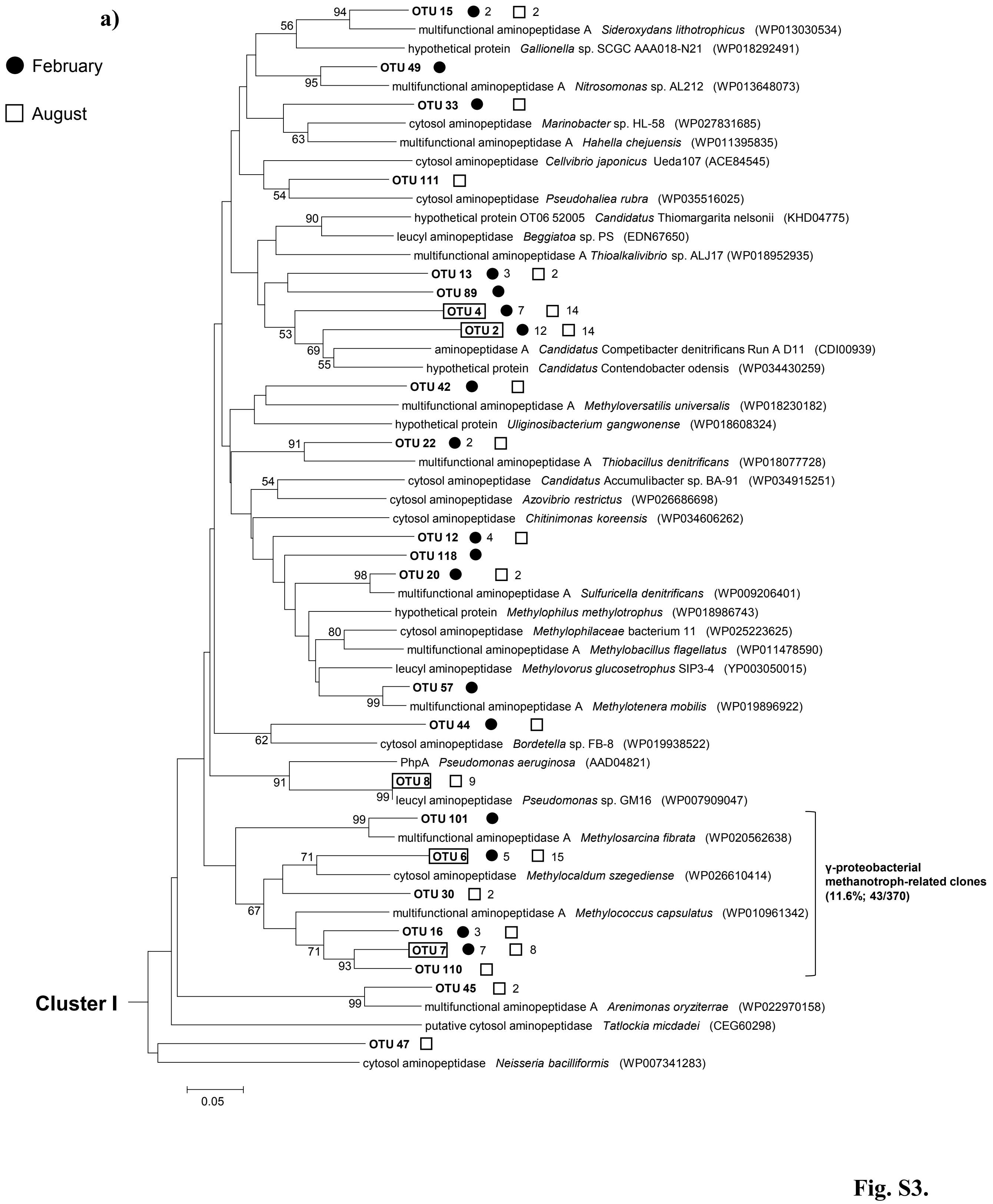
February
August
OTU 15 2 2
multifunctional aminopeptidase A Sideroxydans lithotrophicus (WP013030534)
hypothetical protein Gallionella sp. SCGC AAA018-N21 (WP018292491)
OTU 49
multifunctional aminopeptidase A Nitrosomonas sp. AL212 (WP013648073)
OTU 33
cytosol aminopeptidase Marinobacter sp. HL-58 (WP027831685)
multifunctional aminopeptidase A Hahella chejuensis (WP011395835)
cytosol aminopeptidase Cellvibrio japonicus Ueda107 (ACE84545)
OTU 111
cytosol aminopeptidase Pseudohaliea rubra (WP035516025)
hypothetical protein OT06 52005 Candidatus Thiomargarita nelsonii (KHD04775)
leucyl aminopeptidase Beggiatoa sp. PS (EDN67650)
multifunctional aminopeptidase A Thioalkalivibrio sp. ALJ17 (WP018952935)
OTU 13 3 2
OTU 89
OTU 4 7 14
OTU 2 12 14
aminopeptidase A Candidatus Competibacter denitrificans Run A D11 (CDI00939)
hypothetical protein Candidatus Contendobacter odensis (WP034430259)
OTU 42
multifunctional aminopeptidase A Methyloversatilis universalis (WP018230182)
hypothetical protein Uliginosibacterium gangwonense (WP018608324)
OTU 22 2
multifunctional aminopeptidase A Thiobacillus denitrificans (WP018077728)
cytosol aminopeptidase Candidatus Accumulibacter sp. BA-91 (WP034915251)
cytosol aminopeptidase Azovibrio restrictus (WP026686698)
cytosol aminopeptidase Chitinimonas koreensis (WP034606262)
OTU 12 4
OTU 118
OTU 20 2
multifunctional aminopeptidase A Sulfuricella denitrificans (WP009206401)
hypothetical protein Methylophilus methylotrophus (WP018986743)
cytosol aminopeptidase Methylophilaceae bacterium 11 (WP025223625)
multifunctional aminopeptidase A Methylobacillus flagellatus (WP011478590)
leucyl aminopeptidase Methylovorus glucosetrophus SIP3-4 (YP003050015)
OTU 57
multifunctional aminopeptidase A Methylotenera mobilis (WP019896922)
OTU 44
cytosol aminopeptidase Bordetella sp. FB-8 (WP019938522)
PhpA Pseudomonas aeruginosa (AAD04821)
OTU 8 9
leucyl aminopeptidase Pseudomonas sp. GM16 (WP007909047)
OTU 101
multifunctional aminopeptidase A Methylosarcina fibrata (WP020562638)
OTU 6 5 15
cytosol aminopeptidase Methylocaldum szegediense (WP026610414)
OTU 30 2
multifunctional aminopeptidase A Methylococcus capsulatus (WP010961342)
OTU 16 3
OTU 7 7 8
OTU 110
γ-proteobacterial methanotroph-related clones (11.6%; 43/370)
OTU 45 2
multifunctional aminopeptidase A Arenimonas oryziterrae (WP022970158)
putative cytosol aminopeptidase Tatlockia micdadei (CEG60298)
OTU 47
cytosol aminopeptidase Neisseria bacilliformis (WP007341283)
Cluster I
0.05

Fig. S3.

b)

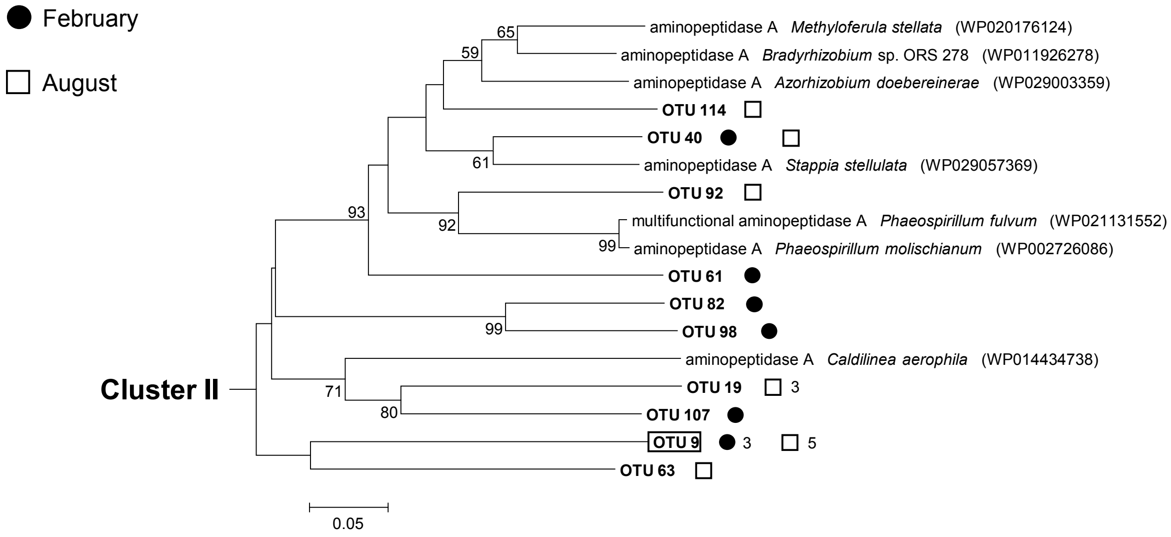

Fig. S3. Continuation.

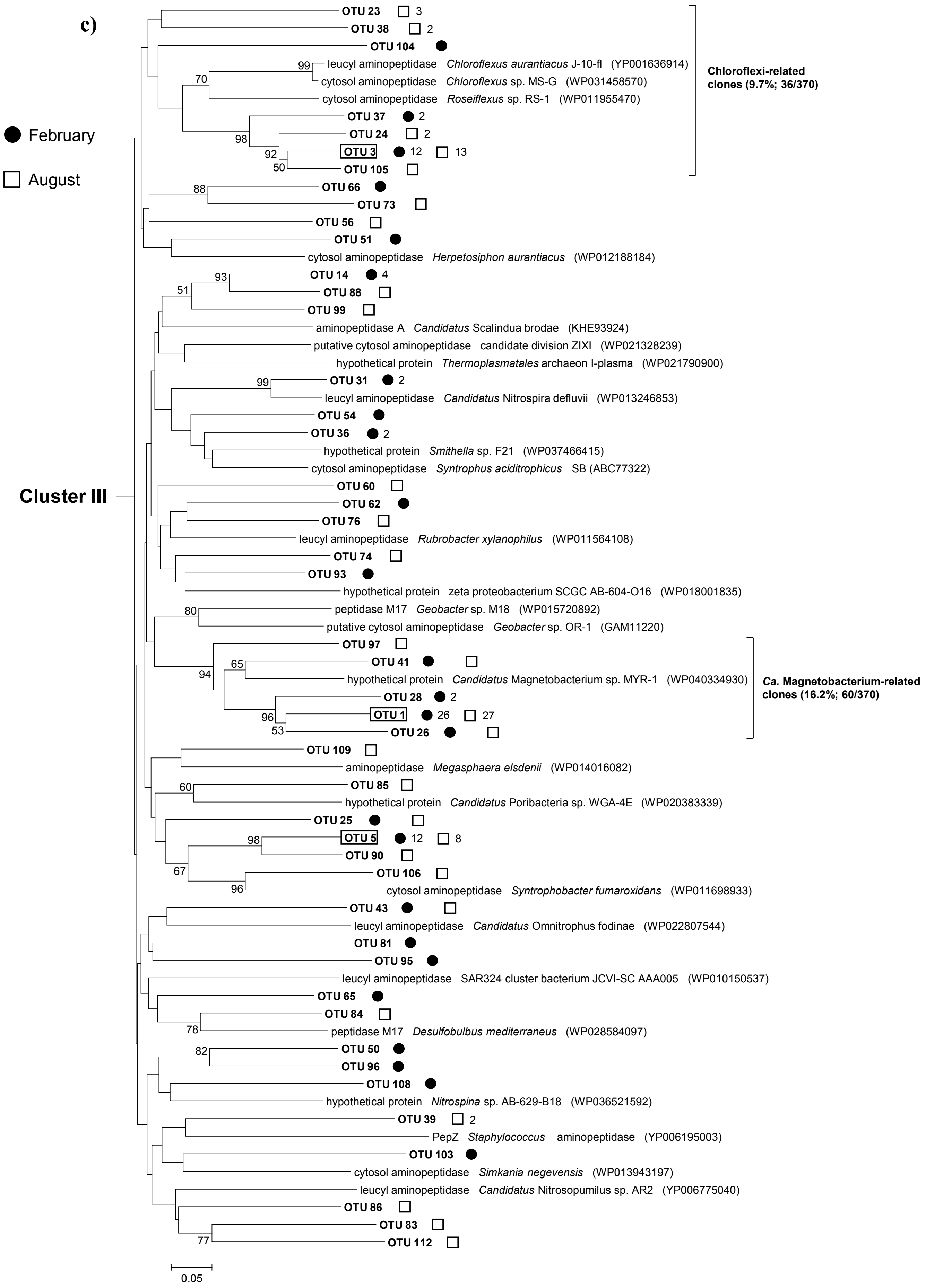

Fig. S3. Continuation.

d)

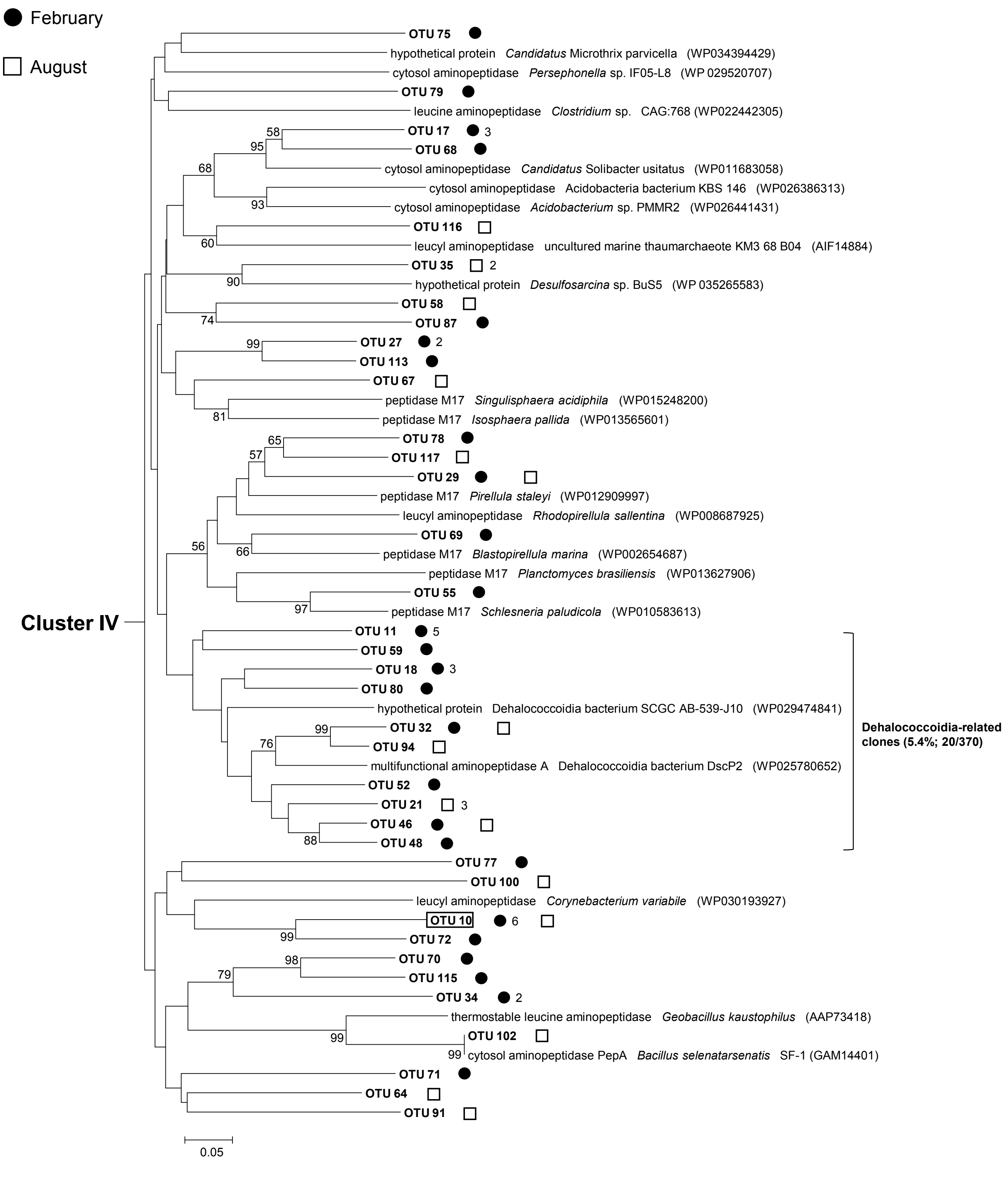

Fig. S3. Continuation.

Reference

1. Chi, M.C., H.B. Huang, J.S. Liu, W.C. Wang, W.C. Liang, and L.L. Lin. 2006. Residues threonine 346 and leucine 352 are critical for the proper function of *Bacillus kaustophilus* leucine aminopeptidase. FEMS Microbiol. Lett. 260:156-161.
